# Efficient prediction designs for random fields

**DOI:** 10.1002/asmb.2084

**Published:** 2014-11-26

**Authors:** Werner G Müller, Luc Pronzato, Joao Rendas, Helmut Waldl

**Affiliations:** a Department of Applied Statistics, Johannes-Kepler-University of LinzLinz, Austria; b Laboratoire I3S, CNRS/Université de Nice-Sophia AntipolisNice, France

**Keywords:** optimal design, Pareto front, empirical kriging, Gaussian process models

## Abstract

For estimation and predictions of random fields, it is increasingly acknowledged that the kriging variance may be a poor representative of true uncertainty. Experimental designs based on more elaborate criteria that are appropriate for empirical kriging (EK) are then often non-space-filling and very costly to determine. In this paper, we investigate the possibility of using a compound criterion inspired by an equivalence theorem type relation to build designs quasi-optimal for the EK variance when space-filling designs become unsuitable. Two algorithms are proposed, one relying on stochastic optimization to explicitly identify the Pareto front, whereas the second uses the surrogate criteria as local heuristic to choose the points at which the (costly) true EK variance is effectively computed. We illustrate the performance of the algorithms presented on both a simple simulated example and a real oceanographic dataset. © 2014 The Authors. *Applied Stochastic Models in Business and Industry* published by John Wiley & Sons, Ltd.

## 1. Introduction

The model underlying our investigations is the correlated scalar random field given by


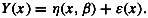


Here, *β* is an unknown vector of parameters in 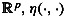, a known function and the random term *ε*(*x*) has zero mean, (unknown) variance *σ*^2^ and a parameterized correlation structure such that **E**[*ε*(*x*)*ε*(*x*^′^)] = *σ*^2^*c*(*x*,*x*^′^;*ν*) with *ν* some unknown parameters. It is often assumed that the deterministic term has a linear structure, that is, *η*(*x*,*β*) = *f*^⊤^(*x*)*β*, and that the random field *ε*(*x*) is Gaussian, allowing estimation of *β* and *θ*={*σ*^2^,*ν*} by Maximum Likelihood. We are interested in making predictions 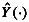 of *Y*(·) at unsampled locations *x* in a compact subset 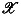 of 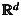 using observations *Y*(*x*_1_),…,*Y*(*x*_*n*_) collected at some design points 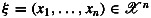. Our objective is to select *ξ* (of given size *n*) in order to maximize the precision of the predictions 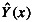 over 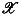. Problems of this type arise in diverse areas of spatial data analysis such as mining, hydrogeology, natural resource monitoring, and environmental sciences; see, for example, 1. This has become the standard modeling paradigm in computer simulation experiments (cf. 2–5), known under the designations of Gaussian process (GP) modelling and kriging analysis.

It is conventional practice that all unknown parameters are estimated from the same data set, but clearly, the classic kriging variance 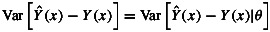 (see Appendix A2) does not reflect the additional uncertainty resulting from the estimation of the covariance parameters; for an early discussion of this issue, see 6. A first-order expansion of the kriging variance for 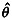 around its true value is used in 7, see also 8 for more precise developments, leading to an explicit additive correction term to the (normalized) kriging variance. This corrected kriging variance, considered in this paper, is given by


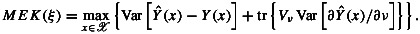
(1)

The design ξ* that minimizes criterion *MEK*(*ξ*) (1) is called empirical kriging (EK)-optimal in 9; see also 10 for another criterion, which is similar in spirit. Mentioned earlier, *V*_*ν*_=*V*_*ν*_(*ξ*,*ν*) stands for the covariance matrix of the estimate of the covariance parameters *ν* and 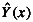 is the posterior mean of *Y*(*x*) given the data at *ξ* = (*x*_1_,…,*x*_*n*_). Note that 
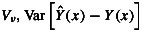
 and 
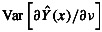
 all depend on *ξ*.

Bootstrap solutions can be found in 11 and 12. The influence of the uncertainty of covariance parameters on the precision of predictions can also be taken into account through a full Bayesian approach, but the computational cost is then much higher than for the standard kriging methodology; see, for example, 13.

In contrast to designs that simply minimize the kriging variance, EK-optimal designs are typically not space-filling, in particular, for small numbers of observations. Unfortunately, maximization of the EK-criterion is computationally demanding, because each evaluation of (1) requires the evaluation of the target function for all points in the candidate set 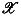, being unfeasible for high-dimensional design spaces as it is often the case with computer experiments. It would thus be useful to have an alternative criterion that can substitute (1) in the optimization procedure while still closely reflecting the actual prediction uncertainty. Note that although minimizing the traditional kriging variance looks equally demanding, one here can usually resort to efficient methods of generating space-filling designs rather than taking a direct approach. Also, the use of an integrated criterion rather than a minimax only alleviates the burden marginally for the corrected kriging variance (but the computational cost is then much reduced when using a spectral approach for the classic kriging variance; see 14).

The paper is organized as follows. In Section 2, we motivate our approach, exploiting the intimate link that should exist between the precision of predictions of the values of the field from a given dataset and the accuracy of the estimates of the process parameters based on the same observations. Section 3 presents the actually new contributions of the paper, proposing two algorithms for identification of EK-suboptimal designs using as surrogates two design criteria related to parameter estimation. Two algorithms for Pareto-optimal design are proposed, both based on the idea of constraining the actual evaluation of *MEK*(*ξ*), see (1), to points in the Pareto front of the surrogate criteria. Finally, Section 4 considers the identification of Pareto-optimal designs for a spatial oceanographic field produced by a biogeochemical mathematical model for the North Sea, and Section 5 draws conclusions on the efficiency and limitations of the approach and suggests topics for future work.

Before presenting the contributions of this paper, it is useful to consider the impact of the correction term in Equation (1) earlier, 
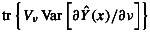
: its influence diminishes as the designs get denser, which happens, for a fixed 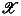, when the number of observations *n* increases. Designs that minimize 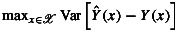 are thus expected to resemble optimal designs for the EK-criterion when *n* is sufficiently large. We illustrate this on a simple example by comparing the behaviors of two greedy strategies for the sequential construction of designs that (*S*_1_) place the next design point at the current maximum of 
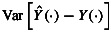
, or (*S*_2_) at the current maximizer of the corrected kriging variance 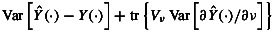.

**Figure 1:**
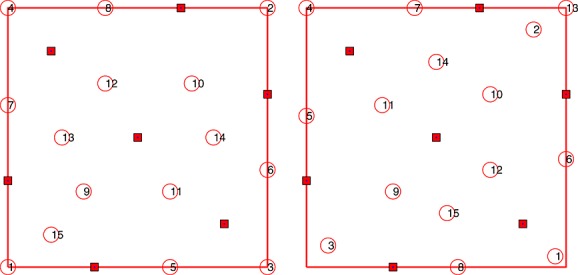
First 15 additional points generated by the greedy strategies *S*_1_(left) and *S*_2_(right) in Example 1.

**Figure 2:**
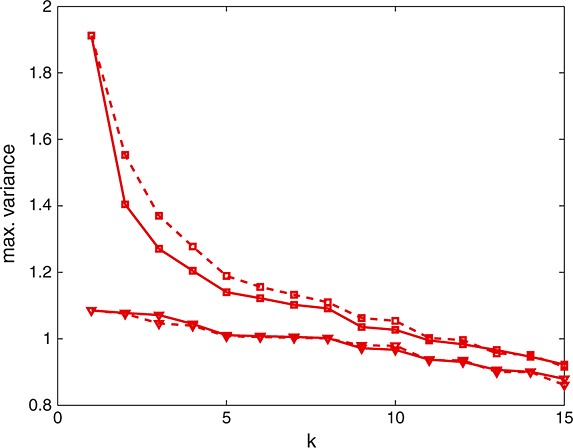
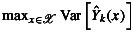
 (triangles) and *MEK*(*ξ*_*k*_) (squares) as functions of *k* for S_1_ (dashed line) and S_2_ (solid line).

*Example 1*Let *σ*^2^=1,*c*(*x*,*x*^′^;*ν*)= exp(−*ν*∥*x* − *x*^′^∥), with *ν* = 7. We assume a constant mean *η*(*x*,*β*) = *β*. For this problem, the design


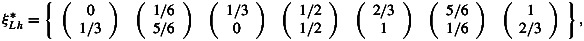
(2)

plotted in Figure 7, left, is simultaneously maximin and minimax distance optimal in[0,1]^2^ in the class of Latin hypercube (Lh) designs with *n* = 7 points; see 15. We consider the sequential augmentation of 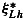 with strategies *S*_1_ and *S*_2_ defined earlier. Denote by 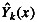 the prediction at *x* for the design 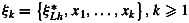. The design obtained by *S*_1_ is space-filling; see 16 for an analysis of its convergence properties in terms of 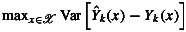 as *k*→*∞*. Figure 1 shows the sequence of design points generated by the two strategies when the design space is 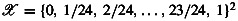, which was chosen to encompass the Lh-designs. Figure 2 shows the evolution of 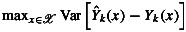 (triangles) and *MEK*(*ξ*_*k*_) (squares) given by (1) as functions of *k*: the dashed line corresponds to *S*_1_ and the solid line to *S*_2_.

All design points added by *S*_1_ tend to fill the design space, whereas the first three points added by *S*_2_ make a compromise between the precision of the prediction with *ν* supposed to be known and the precision of the estimation of *ν*. However, starting with *k* = 4,*S*_2_ tends to be space-filling, too. For 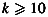, both strategies yield similar values for 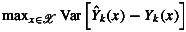 and *MEK*(*ξ*_*k*_), respectively, indicating that the effect of the correcting term in *MEK*(*ξ*_*k*_) becomes negligible as the number of observations increases.

This illustrates the fact that application of the methods presented in this paper is only justified when improvements over space-filling designs are potentially significant. Then, the impact of the correction term added to the classic kriging variance in criterion (1) becomes important, which is the specific setting addressed by this paper. Note that this may depend upon the size of the designs (smaller), the dimension of the problem (larger), and the parameter values. The problem is of practical importance whenever the cost of each observation is large, as it is the case, for instance, in geophysical applications, where it reflects both installation and maintenance of the sensing equipment.

## 2. A relationship inspired by the equivalence theorem

Intuitively, accurate predictions of a spatial field in non-observed sites require good knowledge of the process parameters, and thus designs that optimize prediction-oriented criteria should perform well under criteria that measure estimation accuracy. Such relationships are commonly exploited in the field of design of experiments and run under the heading ‘equivalence theory’. They go back to the celebrated paper by Kiefer and Wolfowitz 17 who, by employing so-called design measures, and for parametric regression models with independent errors *ε*(*x*), established the equivalence of optimal designs for two criteria of optimality, one related to parameter estimation (D-optimality), *i.e*.


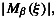


to be maximized, the other related to prediction (G-optimality), that is,


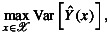


to be minimized.

The analogue to G-optimality for the correlated setup considered here is the EK-criterion (1), which provides a closed-form characterization of prediction uncertainty. Because parameters *β* and *ν*, related to the trend and covariance function, respectively, influence different moments of the process' statistical characterization, they have a remarkably distinct impact on the prediction error. This motivated Müller and Stehlík, see 18 to suggest the use of a convex composition of the two corresponding D-optimality criteria as a surrogate for EK:


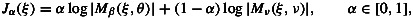
(3)

to be maximized, where


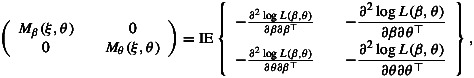


with *L*(*β*,*θ*), the likelihood of *β* and *θ* = (*σ*^2^,*ν*), and *M*_*ν*_(*ξ*,*ν*) in the second term of (3) is the lower diagonal block of *M*_*θ*_(*ξ*,*θ*); see (4).

For the linear model *η*(*x*,*β*) = *f*^⊤^(*x*)*β*, simple computations lead to


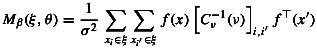


and


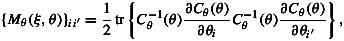


where we used the notation 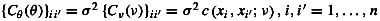. One may note that


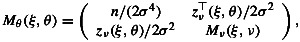
(4)

with


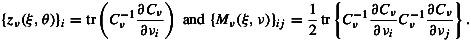


The block *V*_*ν*_(*ξ*,*ν*) of


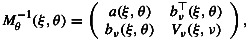


which characterizes the precision of the estimation of *ν* and is used in (1), is given by 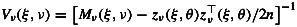 and does not depend on *σ*^2^.

The reason for considering *M*_*ν*_(*ξ*,*ν*) in the definition of *J*_*α*_(*ξ*), Equation (3), instead of the entire matrix *M*_*θ*_(*ξ*,*θ*), is that 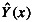 is independent of *σ*^2^, which only intervenes as a multiplicative factor in (1), which thus has no influence on the optimality of a given design for the EK criterion.

The parameter *σ*^2^ is sometimes assumed to be known, and in that case, *V*_*ν*_(*ξ*,*ν*) coincides with 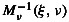. Assumption of knowledge about *σ*^2^ may be motivated by estimability considerations: under the infill design framework, typically not all components of *θ* = (*σ*^2^,*ν*) are estimable, and only some of them, or some suitable functions of them, are micro-ergodic; see 19,20. A reparametrization can then be used; see, for example, 21, with *σ*^2^ set to an arbitrary value. When both *σ*^2^ and *ν* are estimable, there is usually no big difference between *V*_*ν*_(*ξ*,*ν*) and 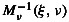. One may refer to 22 for more details on these information matrices and to 23 for computationally efficient implementations for their calculation. We have preferred *M*_*ν*_(*ξ*,*ν*) over 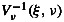 in the definition (3) as it more strongly sharpens the desired balance between space-filling and non-space-filling behaviors, see, for example, 18.

Some efforts have been made to uncover quasi-equivalence relations between optimal designs for prediction and for estimation, compared with 24 or 25. However, it was shown in 26 that a strict equivalence between (1) and (3) does not hold, although optimal designs for one of the criteria tend to perform well under the other, as the example in the succeeding text shows.

**Figure 3:**
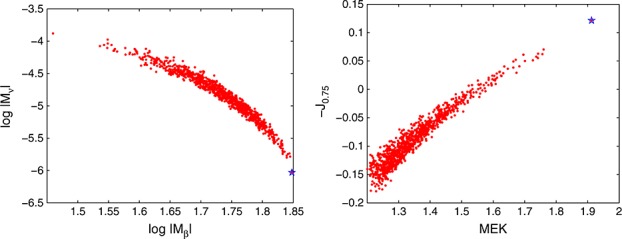
Values of log|*M*_*ν*_(*ξ*,*ν*)| against log|*M*_*β*_(*ξ*,*θ*)| (left) and of −*J*_0.75_(*ξ*) against *MEK*(*ξ*) (right) for 1000 random Lh designs in Example 1 (the star corresponds to 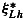 given by (2)).

*Example 1 (continued)*Assume the model in Example 1 and consider 1000 i.i.d. random designs with *n* = 7 points. Each design is a random Latin hypercube (Lh); see, for example, 27, where each component is independently perturbed by the addition of a normal random variable with zero mean and standard deviation 0.1 complemented by truncation to [0,1]. Figure 3, left, shows the values of the two D-optimality criteria log|*M*_*β*_(·,*θ*)| and log|*M*_*ν*_(·,*ν*)| for these 1000 random designs. It is quite apparent that these two criteria are antagonistic. The star in the Figure corresponds to the values of the two optimality criteria for the maximin and minimax distance optimal Lh design 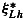; see (2). As anticipated, the space-filling 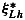 yields a precise estimation of *β* but is extremely poor for estimating *ν*. We also computed, for each of the random designs, the value of the EK criterion. Figure 3 (right) presents the values of −*J*_*α*_(·) for *α* = 0.75 against those of *MEK*(·) for the same set of designs (the evaluation of MEK uses 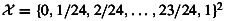). The first thing that we can observe is the good correlation of the two criteria for this choice of *α*. Again, we note that 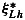 is the worst design for both criteria (they should be minimized). Points in the bottom left corner correspond to designs that are nearly simultaneously optimal for both criteria, confirming the conjecture about the possibility of inferring EK-optimality from the two D-optimality criteria.

However, the correlation between *MEK*(*ξ*) and *J*_*α*_(*ξ*) observed in the example earlier can be much weaker for other values of *α*, and the determination, without evaluating *MEK*(·), of an *α*^⋆^ such that the maximization of 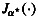 yields a design close to optimality for *MEK*(·) is a difficult open problem. An expression with a structure analogous to criterion (3) can be obtained if we search for the design that minimizes the entropy of the posterior distribution of the predicted field. The comparative analysis of the expressions of the two criteria leads to the conclusion that reasonable values of *α* must be constrained to the interval [0.5,1].

## 3. Pareto-optimal designs

In Section 2, we argued that finding designs *ξ* that minimize the EK criterion (1) should be intimately related to finding designs that optimize a suitable combination of the D-optimality criteria for *β* and *ν*. However, our ability to define a constructive experimental design method based on *J*_*α*_(·) is hampered by the lack of an efficient methodology to select *α*.

In this section, we present two methods that overcome this difficulty and that effectively lead to design algorithms with complexity compatible with applications to real-case scenarios, as the one considered in Section 4. The idea underlying both algorithms is to consider the individual values of the two criteria log|*M*_*β*_(*ξ*,*θ*)| and log|*M*_*ν*_(*ξ*,*ν*)|, and to constrain the candidate set *Ξ* for the minimization of (1) to the set of non-dominated designs for the corresponding multicriteria optimization problem. The algorithms differ in the manner they approximate in this non-dominated solution set. The EK criterion (1) will thus play the role of a preference function for choosing designs in the reduced candidate set *Ξ*.

Other authors have addressed experimental design as a multicriteria optimization problem, constraining the set of possible solutions to those indicated by the corresponding Pareto surface, for example, 28 and 29, where the authors discuss its advantages over the use of scalar ‘desirability functions’ and propose methods to choose amongst the efficient solutions of the Pareto surface. The main new contribution of our paper is the identification of two specific criteria whose set of non-dominated solutions is a relevant (small) candidate set for optimization of the EK variance.

The set Ξ of non-dominated (or Pareto-optimal) designs for the multiple objective optimization problem defined by log|*M*_*β*_(·,*θ*)|, and log|*M*_*ν*_(·,*ν*)| is defined by


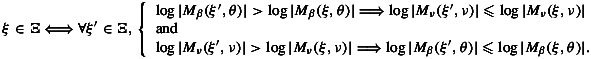


The solid line in Figure 8 is an example of a Pareto surface for simultaneous maximization of the two criteria.

For *K* functions *φ*_*i*_(·) to be maximized with respect to some variables *ξ* and taking values that vary continuously in *K* intervals *I*_*i*_, the Pareto surface, or Pareto front, is in general, a (*K* − 1)-dimensional bounded surface included in 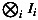. In our case, *K* = 2 and the Pareto surface 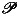 reduces to a bounded curve—to a finite subset of a curve when 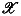 is finite. Let *P*(*ℓ*) = (*C*_*β*_(*ℓ*),*C*_*ν*_(*ℓ*)) be a parametrization of the Pareto surface, with *ℓ* denoting an index for its points. We denote by {*ξ*}(*ℓ*) the set of designs that map to point *P*(*ℓ*) in 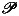.

In what follows, we consider only designs constructed over a finite subset 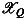 of the compact design space 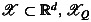 having *Q* elements. 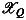 can be, for instance, a regular grid, with *Q* growing with *d* like *q*^*d*^ for some *q*, or the points of a low-discrepancy sequence; see, for example, 30. Also, the maximization over 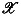 in (1) will be replaced by maximization over a finite subset 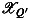 of 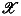 with *Q*^′^ elements. In general, we shall omit the index *Q* and simply write 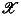 for 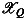. Unless otherwise stated, we shall take 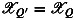, but other choices are possible. Also, in this paper, we only consider designs without replications.

### 3.1. Minimizing *MEK*(*ξ*) over the set of Pareto-optimal designs

In general, {*ξ*}(*ℓ*) is not a singleton and *MEK*(·) is not constant over this set. Moreover, the minimizer of *MEK*(·) over 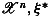 does not generally belong to some {*ξ*}(*ℓ*). The minimization of *MEK*(·) over 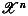 is therefore not equivalent to the minimization of *MEK*(·) over the set of Pareto-optimal designs. However, if our belief that the two parametric estimation criteria log|*M*_*β*_(·,*θ*)| and log|*M*_*ν*_(·,*ν*)| yield good surrogates for the EK criterion is valid, then (*i*) the variation of *MEK*(·) over each set {*ξ*}(*ℓ*) should be much smaller than its variation across distant points in the Pareto surface 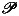 (this fact has been checked numerically on simple examples), and (*ii*) the minimum of *MEK*(·) over the Pareto-optimal designs should approach the minimum of *MEK*(·) over 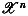.

The method proposed in this section is based on the identification of a finite set of Pareto-optimal designs *Ξ*_*P*_, the final design being obtained by minimizing *MEK*(·) over this reduced set:


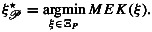


**Figure 4:**
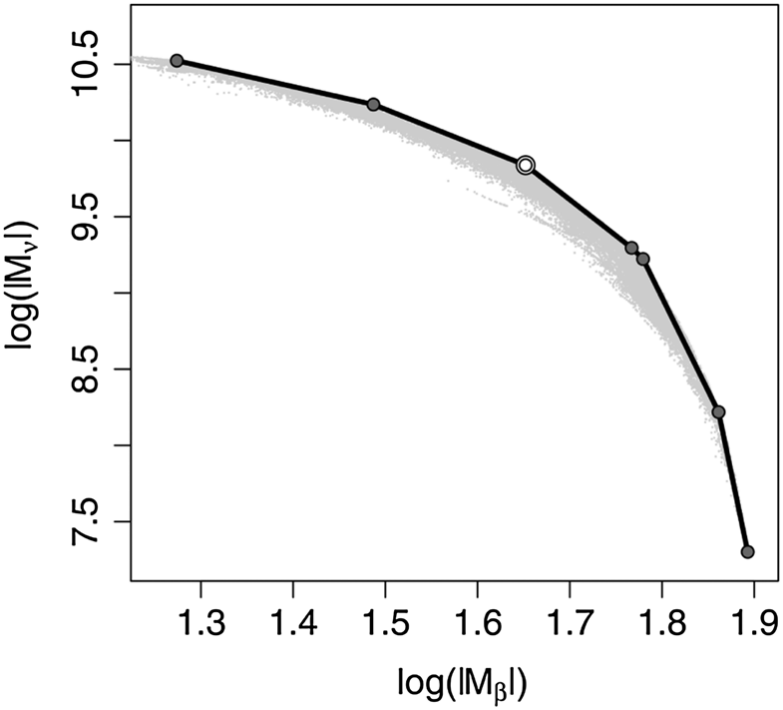
Sampled points (in grey) for generating the Pareto surface (black); seven points lie on the convex hull, the one in white being selected.

Because the Pareto surface is the set of maxima of all scalar functions monotone in each criterion, we can construct a finite set of candidate designs Ξ_*P*_ by optimizing *J*_*α*_(·) for a finite set of values of *α*. Because the maximization of *J*_*α*_(·) can only give points that belong to the convex hull of 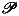, we may thereby miss some regions of the Pareto front, but we found on numerical examples that the effect is marginal, see, for example, Figure 4.

The optimization of *J*_*α*_(·) for fixed *α* is carried out using a simulated annealing (SA) algorithm; see, for example, 31–33. In the examples in the succeeding text, the following implementation of the SA algorithm has been used (remember, we want to maximize *J*_*α*_(·)):

**Step 0** *Initialization*. Set initial temperature *T*_0_ and cooling factor *r*∈(0,1).Draw initial design *ξ*_0_∼*p*_0_(*ξ*)∝1,*e*_0_=*J*_*α*_(*ξ*_0_).Set current best solution 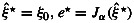.Set *k* = 0.
**Step 1** *Generate candidate*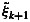 by random perturbation of 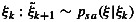.
**Step 2** *Perform a local optimization* of *J*_*α*_ around 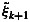:
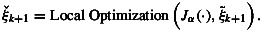
**Step 3** *Update best solution*. Let 
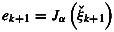
. If *e*_*k* + 1_>*e*^⋆^, then 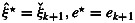.
**Step 4** *Random acceptance*. If *e*_*k* + 1_>*e*_*k*_, set 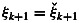. Otherwise, set
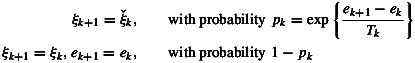
**Step 5** *Temperature update*. If *ξ*_*k* + 1_=*ξ*_*k*_ (no change has been made in step 4), update the temperature according to a geometric cooling scheme: *T*_*k* + 1_=*rT*_*k*_.
**Step 6** *Stopping condition*. If *k* = *N*_*max*_ stop; otherwise *k*←*k* + 1, return to step 1.

Throughout the algorithm, we keep track of the best solution found, which is eventually reported as 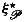. It is also expedient to start the algorithm with a space-filling design *ξ*_0_ to quickly weed out the cases for which our method is obviously unnecessary.

Like most random-search algorithms, under assumptions that are easily satisfied, the SA algorithm earlier allows us to reach an arbitrary neighborhood (in terms of criterion value) of a global maximum of *J*_*α*_(·) in a finite number of iterations almost surely; see, for example, 31. However, convergence may be slow and the risk of stopping the algorithm well before reaching some reasonable neighborhood of an optimal solution cannot be neglected.

The random perturbation *p*_*sa*_(*ξ*|*ξ*_*k*_) in step 1 consists in the replacement of two randomly chosen points (*x*_*i*_,*x*_*j*_) of *ξ*_*k*_ by two points uniformly drawn (without replacement) from 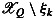. A more generalized version could replace up to *n* points.

In step 2, local optimization (*J*_*α*_(·),*ξ*) is a procedure that performs iterative optimization of *J*_*α*_(·), starting from design *ξ*. Our implementation assumes that 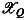 is a regular rook-type grid (the clique *V*_*x*_ of point 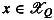 is defined as the set of its North, South, West, East neighbors in 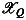).

*Local optimization*(*J*_*α*_(·),*ξ*)Do { Set *ξ*_0_=*ξ* Set *J*_0_=*J*_*α*_(*ξ*_0_),*J* = *J*_0_. For all *x*_*i*_∈*ξ*_0_ (scan all points in *ξ*) For 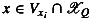 (consider replacement by all points in the clique of *x*_*i*_) Set 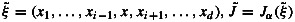 If 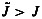 set 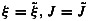} while *J* > *J*_0_Return(*ξ*_0_)

*Example 1 (continued)* We illustrate now, for the process introduced in Example 1, the application of this method for finding seven-point designs for prediction over the finite design space 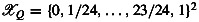.

Figure 4 shows the seven distinct values on the Pareto surface obtained by maximization of *J*_*α*_ for 11 values of *α* uniformly spread in [0.5,1]. The following parameters were used for the SA algorithm: *T*_0_=0.6,*r* = 0.93,*N*_*max*_=5000. Tests over a large number of executions of the SA led to no noticeable variations of the Pareto-front in Figure 4.

*MEK*(·) was subsequently computed for the seven Pareto-designs and 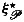 selected as the best one:


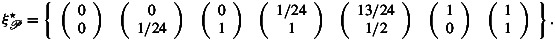
(5)

**Figure 5:**
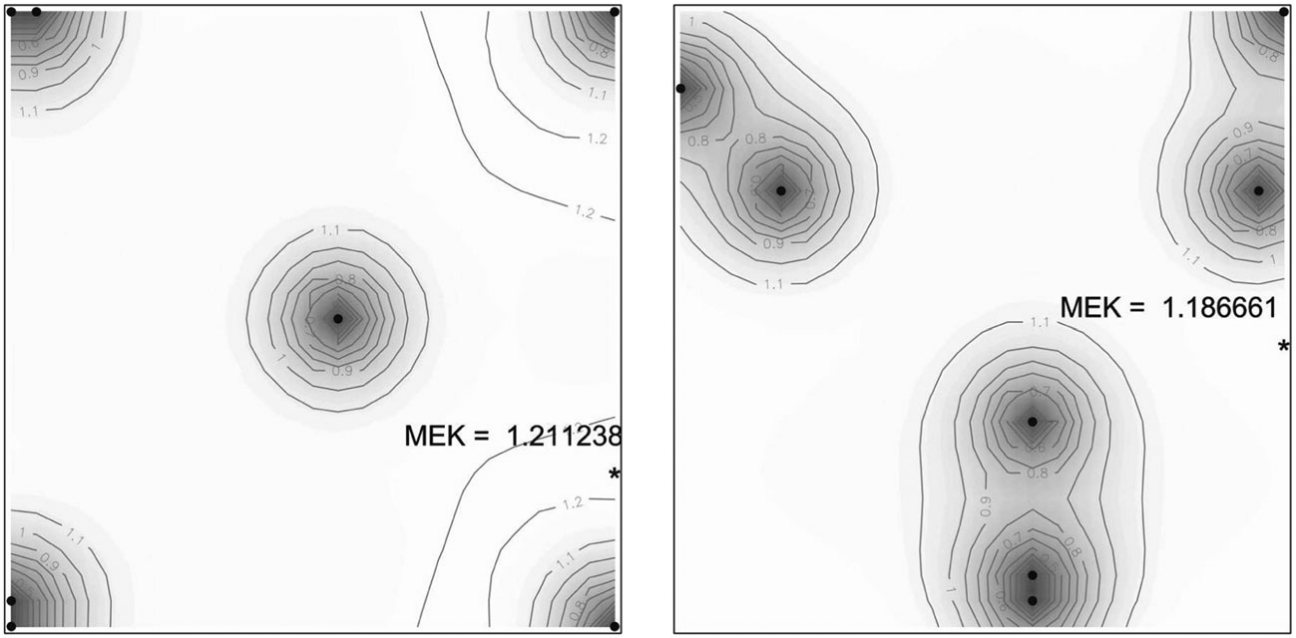
Corrected kriging variance for the Pareto-optimal design (left) and the empirical kriging optimal design (right). Black dots indicate the design points.

In Figure 5, left, we present a contour plot of the corrected Kriging variance for 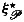. In the plot, the black dots indicate the design points, at which the variance is zero, the asterisk indicates the location of the maximum.

We also searched directly for the optimal *EK* design *ξ*^⋆^ by optimizing *MEK*(·) using the SA algorithm. The much higher computational complexity of criterion evaluation imposed in this case constraining the maximum number of iterations of the SA algorithm to *N*_*max*_=2000. The optimal design obtained is shown in Figure 5 (right) along with the corresponding surface of corrected Kriging variance. The effectiveness of the method can be appreciated by computing the efficiency of the Pareto-optimal design 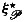 with respect to the optimal design *ξ*^⋆^, which is in this case 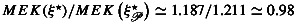.

**Figure 6:**
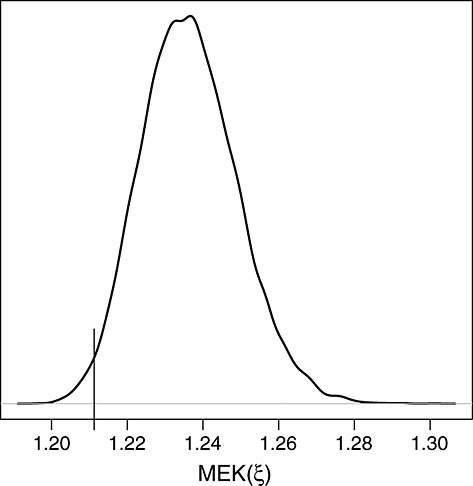
Empirical distribution of empirical kriging minima for 10 000 random sets of seven designs; vertical bar indicates 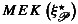.

Notice that the construction of 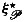 only required seven evaluations of the expensive criterion *MEK*(·). For completeness, we also simulated 10 000 random sets of seven designs 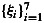 and computed min*iEK*(*ξ*_*i*_) for each. The empirical distribution of these minima is given in Figure 6. It shows that 98% of the random designs generated with the same effort as ours lead to a corrected kriging variance larger than the one obtained using 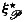.

### 3.2. A simplified exchange algorithm

The method proposed in this section is based on an idea suggested in 15. Like the algorithm earlier, it makes use of the Pareto front, but, in contrast to it, is deterministic, stops after a finite number of iterations when 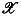 is finite, and therefore cannot provide any guarantee of asymptotic convergence. We call *exchange* the substitution of one point 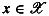 for one point *x*_*i*_ of the current design *ξ*. For any given design *ξ* with *n* distinct points in 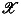, there are thus *n* × (*Q* − *n*) possible exchanges with *Q* the number of elements in 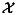. The algorithm starts with an arbitrary design, for example, space-filling, and exchanges one point at a time; only exchanges corresponding to non-dominated solutions for the two criteria log|*M*_*β*_(·,*θ*)| and log|*M*_*ν*_(·,*ν*)| are retained for the evaluation of *MEK*(·); the best among them gives the design carried to the next iteration.

**Step 0** *Initialization*. Choose a space-filling design *ξ*_0_ with *n* points (*e.g*., a Lh design), compute 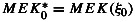, set *k* = 0.
**Step 1** *Construction of the Pareto front*. Construct the *N*_*k*_ designs 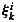 corresponding to all possible exchanges for *ξ*_*k*_ and compute the associated values of 
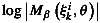
 and 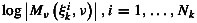; construct the subset Ξ_*k*_ of designs 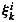 that correspond to non-dominated solutions for log|*M*_*β*_(·,*θ*)| and log|*M*_*ν*_(·,*ν*)|.
**Step 2** *Evaluation of the EK-criterion*. Compute 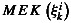 for all 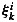 in Ξ_*k*_.
**Step 3** *Design update*. If 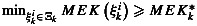, stop;otherwise set 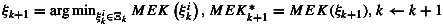, return to step 1.

At step 1, *N*_0_=*n* × (*Q* − *n*) exchanges are considered at first iteration, but *N*_*k*_=(*n* − 1) × (*Q* − *n*) for 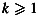, because we do not need to consider the exchange of the same point of *ξ* for two consecutive iterations (although this was not used for the example in the paper, one may also restrict the exchange of *x*_*i*_ with points in some neighborhood, which significantly reduces *N*_*k*_). Note that not all 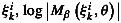 and 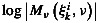 have to be stored because the set of non-dominated solutions Ξ_*k*_ can be constructed iteratively. A further simplification is obtained by restricting Ξ_*k*_ to designs that correspond to points on the convex hull of the Pareto front (which can also be constructed iteratively). A continuation of Example 1 gives an illustration.

**Figure 7:**
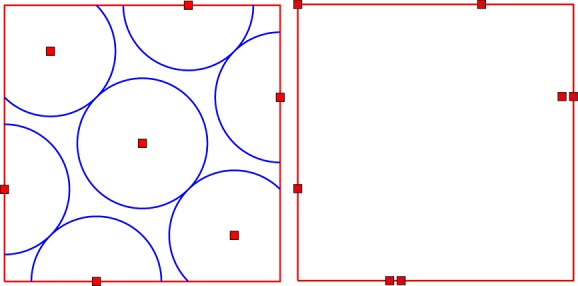
Latin hypercube design 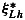 (2) (left)—the circles have radius 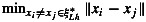
—and design *ξ*_4_ (6) (right).

**Figure 8:**
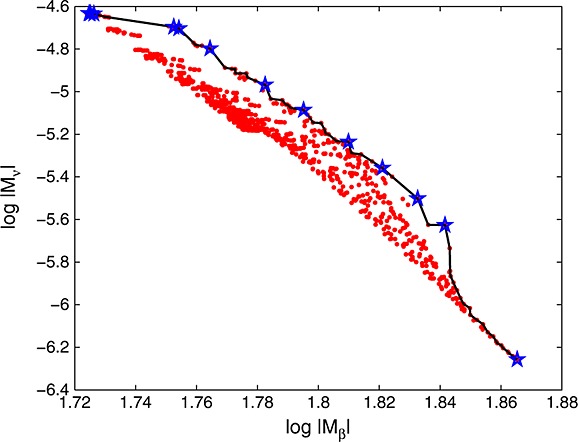
Values of 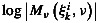 against 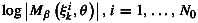, at iteration 1 of the simplified exchange algorithm in Example 1: the stars correspond to points on the convex hull of the Pareto front, which is indicated by the solid line.

*Example 1 (continued)*We, again, restrict 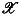 to the 25 × 25 grid of points with coordinates in the set 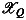. Note that this set contains the design 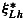 given by (2), which is chosen as initial design *ξ*_0_ (with *MEK*(*ξ*_0_)≃1.9124). The algorithm earlier, with Ξ_*k*_ given by all points on the Pareto front stops after three iterations and returns a design with *MEK*(*ξ*_3_) = 1.2060, requiring 967 evaluations of the EK-criterion. When Ξ_*k*_ is restricted to the points on the convex hull of the Pareto front, the algorithm stops after four iterations and returns the design


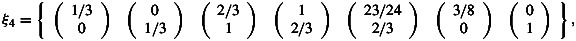
(6)

see Figure 7 (right) with *MEK*(*ξ*_4_)≃1.2080. Figure 8 shows the values of 
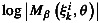
 and 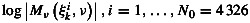, at the first iteration of the algorithm. There are 296 non-dominated points on the Pareto front (in solid line), but only 15 points (indicated by stars) on its convex hull. The restriction of Ξ_*k*_ to those points thus reduces the computational cost significantly: the EK-criterion (1) is only evaluated 45 times in total when the algorithm stops. Note that although this is six times more often than the procedure of Section 3.1, it gives a slight improvement of the criterion and is still considerably quicker than the SA procedure.

## 4. Design of sensors for an oceanographic field

We consider in this section application of the proposed design methodology to a case of practical interest, where the goal is to design a network of fixed observation sensors for an oceanographic field using outputs of a formal (numerical) model.

**Figure 9:**
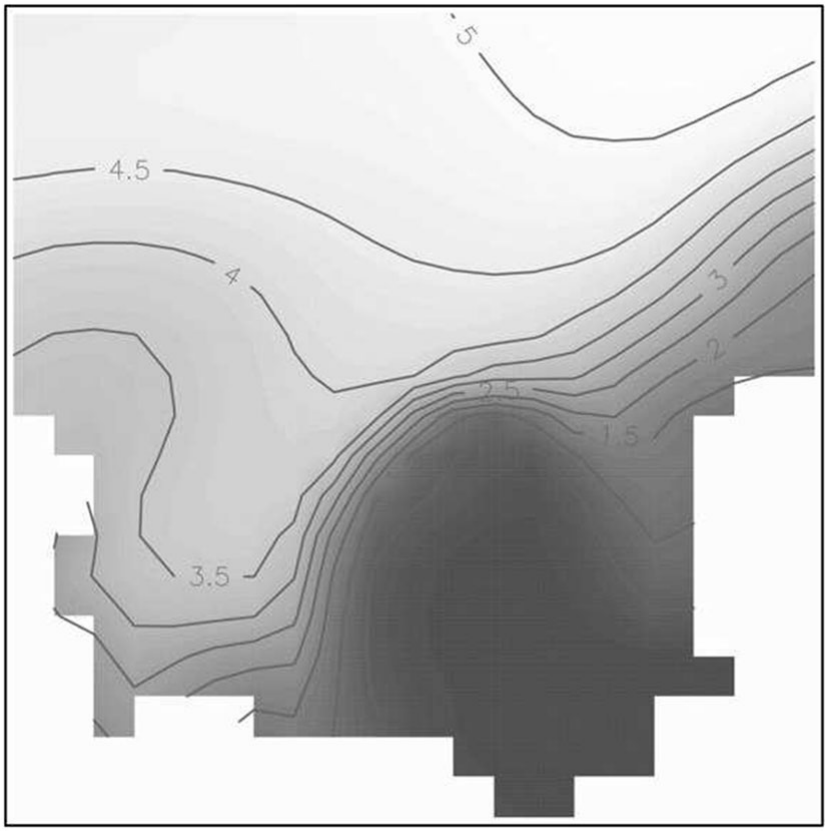
Ammonium field over the region of interest.

The data used in this study were made available through a collaboration with the institute MUMM, a department of the Royal Belgian Institute of Natural Sciences. It consists of snapshots of the output of the biogeochemical oceanographic model MIRO&CO 34, run to simulate the evolution of inorganic and organic carbon and nutrients, phytoplankton, bacteria, and zooplankton with realistic forcing conditions. The outputs available cover 5 years, with a periodicity of 1 week, yielding a total of 258 maps. The model covers the entire water column of the Southern Bight of the North Sea, but in the study presented here, we concentrate on a horizontal (sea surface) grid of 21 × 21 points corresponding to the bay of the Seine river. We remark that the area of interest has a realistic geometry, in particular, it is not convex as it can be seen in Figure 9.

MIRO&CO results from the integration of four modules describing: (i) the dynamics of phytoplankton, (ii) zooplankton, (iii) bacteria and dissolved/particulate organic matter degradation and (iv) nutrient (nitrate (NO3), ammonium (NH4), phosphate (PO4), and dissolved silica (DSi)) regeneration in the water column and the sediment. We limited our study to design of a network for observation of the distribution of NH4, one realization of this field is illustrated in Figure 9, more precisely to identify the seven-point design that would enable the best extrapolation of the NH4 field to all grid points. The data simulated by the model over the entire grid has been used as a proxy to evaluate actual prediction uncertainty.

The problem addressed in this section is thus representative of the design of networks of fixed oceanography stations with limited size based on the predictions of (complex and numerically expensive) computer models, and can be found in many other application areas, such as meteorology, agriculture, air quality, and pollution surveillance. Two possible future extensions of practical interest would be (i) the identification of the best observation network for a *vector* field, for instance, for the observation of several components of the output of model MIRO&CO, for instance NH4 *and* zoo plankton; and (ii) exploitation of the temporal correlation of the observed fields. Although the latter can be, at least formally, handled using the same approach that is used here, by considering time as an additional coordinate and model both spatial and temporal trend and correlation, the former requires a deeper modification of the framework used in this paper, calling for more complex kriging algorithms.

In order to have a realistic evaluation of the extrapolation errors that can be expected in realistic conditions, we split the available model outputs in two sets. One set (of size *M*_*L*_) is used for learning the parameters of the statistical model, on the basis of which the design is defined. The other set, of size *M*_*T*_, is reserved for assessing the actual extrapolation performance, by computing the errors affecting the extrapolation of the values at the design points to other grid points, as explained in 4.3 in the succeeding text. We believe that for realistic models—like those that are currently used for operational meteorology or oceanography, like MIRO&CO—the correlation structure of model predictions closely reflects the local correlation structure of the true fields, and thus that our error evaluation is representative of the deviations between the extrapolated and the real fields that can be expected in reality.

The study compares the performance of the following designs:

- the optimal design for the corrected EK criterion *ξ*^⋆^;
- the SA-optimization based Pareto design 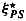 (Section 3.1);
- the deterministic local Pareto optimisation design 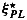 (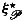 in Section 3.2); and
- the optimal simple Kriging-space-filling design 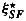.

### 4.1. Model fitting

Because our design criteria depend on the process model, we started by fitting a GP model to the outputs of the numerical model available. As explained earlier, only a part of the available data is used for model learning. As the available data spans several years, model learning will be based on the entire first year (1993) to cover the expected seasonal variations, the rest (1994–1997) being used for performance assessment.

Let *M*_*L*_=49 denote the size of the learning set, and 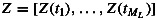 the corresponding set of model outputs, each *Z*(*t*_*j*_) gathering the values of the NH4 field at time (i.e., week) *t*_*j*_ over the 21 × 21 grid of analysis. We assume that the snapshots are all statistically independent realisations of the same GP. *Z*(*x*,*t*_*j*_) is the NH4 value at time *t*_*j*_ at site 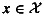, which is modeled by


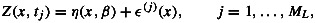
(7)

where the *β* are unknown deterministic coefficients and the Gaussian fields *ε*^(*j*)^(*x*) are statistically independent realisations of the same GP. Using Maximum Likelihood, we fitted model (7) with linear trend and Matérn correlation function


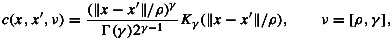


where *K*_*ν*_(·) is the modified Bessel function of the second kind of order *ν*, to the 49 field snapshots from 1993:


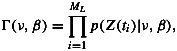


obtaining


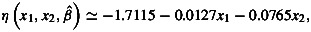



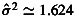
, range parameter 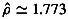, and smoothness parameter 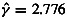. All computations were performed by using the function *likfit* of the R-package *geoR* (35, details of its usage can be most conveniently found in 36. We have also employed the built in correction for geometric anisotropy, which amounts to rotating and stretching the coordinates by multiplication with


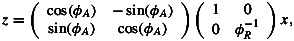


yieldingratio 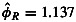 and angle 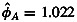. For simplicity, these parameters were fixed for later evaluation purposes as well as *γ* set to 5/2, which gives the much simplified


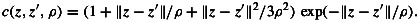
(8)

### 4.2. Finding the optimal designs

**Figure 10:**
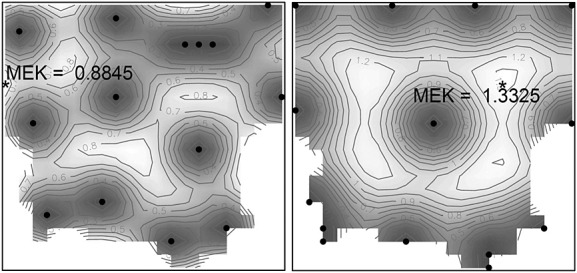
Empirical kriging variance contour maps for *ξ*^⋆^ (left) and 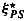 (right).

We have chosen *n* = 14 throughout this example as this was the largest design size for which the Pareto-optimal designs were still superior to the space-filling with respect to the EK-criterion. The Pareto-optimal design 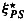 for the aforementioned model in the region of analysis has then been found by the method presented in Section 3.1, where 18 distinct points were identified on the convex hull of the Pareto-surface. The parameters of the SA algorithm were set as in Example 1, again, the algorithm was started from a random initialization; the minimal EK variance was identified for a quite high *α* = 0.99. In Figure 10, we plot the corrected kriging variance for this design (right) and by direct optimization of the EK criterion (left), overlaid with the corresponding optimal designs (indicated by the black dots). We can see that while the Pareto-optimal design distributes the sampling points mainly along the boundary of the region of analysis, the EK-optimal design contains several points in the interior of the design space, and is able to keep the corrected kriging variance at lower levels *MEK*(*ξ*^⋆^) = 0.8845 versus 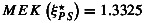.

**Figure 11:**
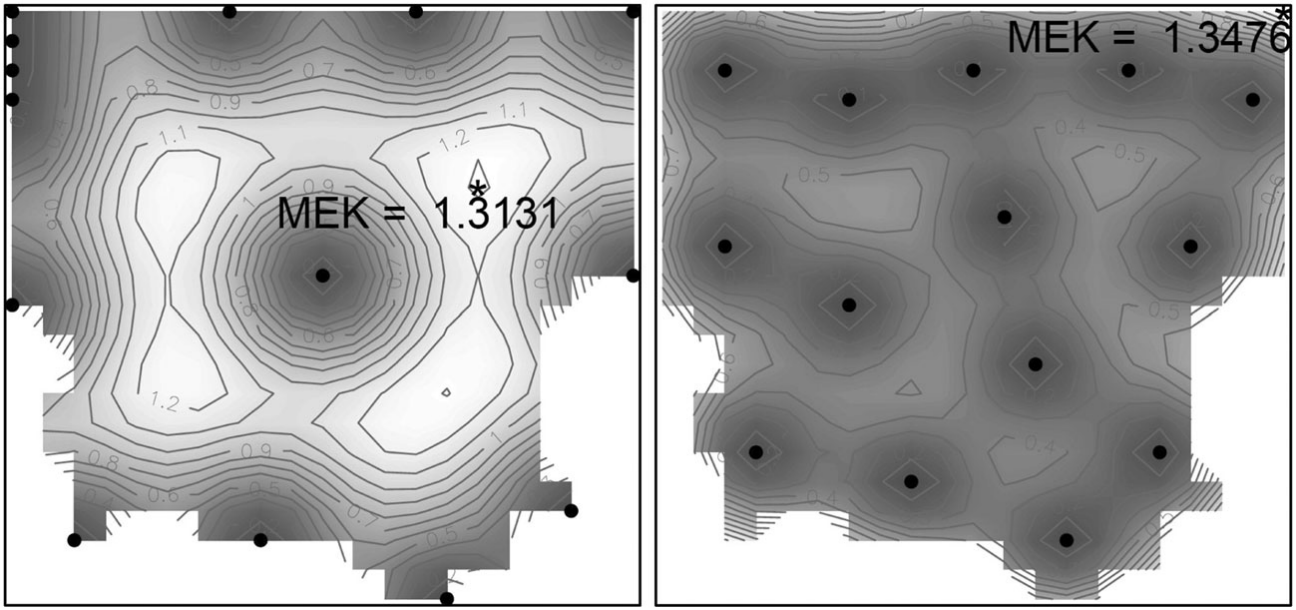
Empirical kriging variance contour maps for 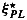 (left) and 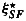 (right).

We likewise employed our sequential algorithm to yield 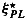 albeit requiring 12 iterations with a total of 78 evaluations of the EK-criterion, yielding 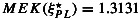. For comparison, we have also calculated a 14-point space-filling design 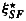 utilizing a minimax distance criterion, which is conveniently implemented as function *cover.design* in the R-package *fields* 37, giving 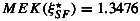 (a depiction of this design is given in Figure 11 (right)).

A comparison of computing times between the SA-based Pareto algorithms with direct optimisation of the EK-criterion has been carried out. Using a standard computer (with Intel Core i5 520M / 2.4 GHz, Dual-Core) a ratio of approximately 1 : 200 in favor of the SA-based algorithm has been observed for the small example in Figure 11. We expect this ratio to improve as the complexity of the problem (the dimension of the input space and/or the size of the design) increases.

### 4.3. Performance evaluation

We finally evaluated the performance of our quasi-optimal designs by assessing the extrapolation error over the *M*_*T*_=196 fields in the test set, which were not used for model learning.

For each snapshot *Z*_*j*_=*Z*(*t*_*j*_) in the test set 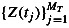, we have firstly computed the corrected kriging variance for the fitted model and its maximum


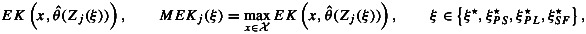


where 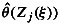 denotes the ML estimator of *θ* = (*σ*^2^,*ρ*), see (8), obtained from the data *Z*_*j*_=*Z*(*t*_*j*_) at the design points in *ξ*. It turns out, however, that these empirically derived EK values are very sensitive to the individual weekly observations and vary strongly, particularly for the space-filling designs, due to the small design size. Their respective medians over the test set, however, well reflect the inferior performance of the space-filling design as they yield 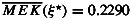, 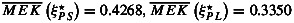, and 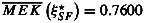, respectively.

Secondly, we computed the maximum squared error over the grid of analysis


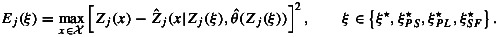


**Figure 12:**
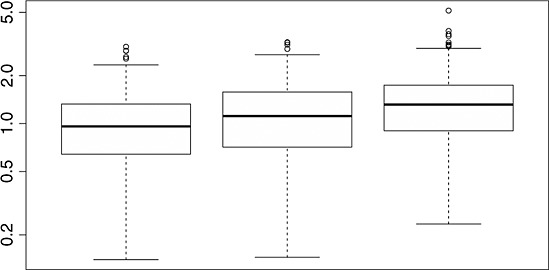
Boxplots of the ratios of *E*_*j*_ versus *E*_*j*_(*ξ*^⋆^) on a log scale for 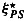 (left), 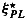 (middle) and 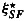 (right).

In Figure 12, we present the ratios of *E*_*j*_ for 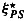 (left), 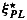 (middle), and 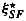 (right) versus *E*_*j*_(*ξ*^⋆^) on a log scale. Here, the two Pareto based designs seem to be comparable with the EK-optimal design, whereas the space-filling design performs clearly worse.

Finally, we compute an indicator of the consistency of the design criteria, by computing the empirical mean-square error over the *M*_*T*_ realisations in the test set:


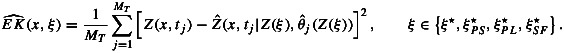


Those empirical counterparts of our original design criterion can be compared for the four employed designs. Although the EK-optimal eventually yields 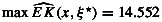, we get even better values for 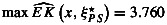 and 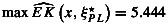, whereas 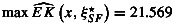 is clearly the worst. All of these maxima are realized at the same location close to the harbour of Le Havre, indicating that the model fails to predict the high values of the field in the South-East small region, whose correlation structure strongly departs from the smoother variation in the open sea region, invalidating the predictions of the kriging variance. Note that these errors are strong even for the EK optimal design where a design point is located near that region. Another factor that may be affecting performance of the predictors in this region is related to the fact that the region of analysis is not convex, and thus the use of a covariance model based on simple Euclidean distance, like the Matérn model, cannot capture the internal structure of the water mass, which is confined by the region bathymetry.

Note that it may be more adequate to address the spatio-temporal nature of the data by allowing the parameters in the model to vary. For simplicity, we refrain from introducing that here, although due to lower estimates for *σ*, we expect further improvements of the performance of the Pareto-based designs. Furthermore, various other refinements are certainly possible that will be treated in future work. We are confident that the present exposition achieves the intended illustrational purpose well.

## 5. Conclusions

This paper proposes methods for identification of designs quasi-optimal for the corrected kriging variance in the context of prediction of spatial Gaussian fields. The criterion, also known as EK criterion, that takes into account the increased variance due to limited accuracy of the estimates of the covariance of the GP, is especially important when this uncertainty is expected to make the corresponding optimal designs depart from space-filling.

Two methods are presented, both based on using design criteria for the estimation of process parameters (related to the trend and to the covariance of the random term). These design criteria are to be simultaneously optimized, as surrogate criteria for the EK-minimization. They offer increased efficiency compared with direct optimization of the corrected kriging variance, by limiting the evaluation of the numerically expensive EK-criterion to the Pareto-front of the two criteria. The methods differ on how the Pareto-surface is determined: whereas one relies on the use of stochastic optimization (SA) to sample the Pareto-front by optimizing distinct convex combinations of the two design criteria, the second is deterministic and iteratively approaches this surface. They have characteristics that are dual in some sense: whereas in the first the number of sampled points of the Pareto surface is fixed by the user (who sets the number of convex combinations that are optimized), in the second, the number of evaluations of the EK criterion is not fixed in advance. The price paid for the controlled complexity of the former is a potentially poorer sampling of the Pareto surface, leading eventually to a larger EK-value for the chosen design.

The paper illustrates the two methods both in a simple simulated model and also on a real application with an oceanography data set. The results obtained show the validity of the approach underlying the two algorithms, which are able to identify designs that are close to optimal efficiency, and yield prediction variances that may be significantly lower than it would be possible using standard space filling designs. Of course, as we remark in the introductory sections of the paper, efforts to optimise the EK criterion should be limited to those situations where cost of observations is large and the impact of the estimation of the covariance parameters cannot be neglected. In these cases, the methods proposed here offer a cost-effective alternative to the prohibitive direct optimisation of the relevant EK-criterion. Other approaches, based on complementing a design optimal for the estimation of the trend parameters with a design optimal for the estimation of the covariance parameters, are under current investigation.

## Appendix A. Information matrix and empirical kriging (EK) variance for the Matérn covariance function

We analyze the model *Y*(*x*) = *f*^*T*^(*x*)*β* + *ε*(*x*) with Gaussian *ε*(*x*) with zero mean and Matérn isotropic covariance (cf. e.g., 19)

where *d*_*i**j*_=∥*x*_*i*_−*x*_*j*_∥,Γ(·) is the gamma function, *K*_*γ*_(·) is the modified Bessel function of the second kind with order *γ* and *ν* = (*ρ*,*γ*)^*T*^ are the non-negative covariance parameters.

### A.1. Information matrix for the variance-covariance parameters

Let *θ* = (*σ*^2^,*ν*^*T*^)^*T*^ be the variance-covariance parameters of the Matérn covariance function. Then, the information matrix for *θ* and a design *ξ* = (*x*_1_,…,*x*_*n*_) is given by (4). That means, we have to compute the derivatives

(A.1)

with *C*_*ν*_ simplified to .

*Derivative with respect to**ρ**:*

The computation of the derivative with respect to *ρ* is straightforward. We just have to apply the product rule using  (see 38) and then apply the following Bessel function identity (see 39).

(A.2)

This gives

*Derivative with respect to the order**γ**:*

The computation of the derivative with respect to *γ* is more complicated. Although we do not utilize it in the paper, we give it here for completeness thus allowing for a more refined version of the application. First, we have to apply the product rule using the polygamma function of order 0, . Finally, we again have to apply the identity (A.2)). We obtain

For the derivative of the modified Bessel function of the second kind, we have to compute

where *I*_*γ*_(·) is the modified Bessel function of the first kind with order *γ* (see 38,39).

### A.2. Empirical kriging variance for the Matern covariance function

In order to find EK-optimal designs, we have to minimize the design space maximum of the corrected kriging variance (see Equation (1))

(A.3)

Here,  is the kriging prediction for design *ξ* at point . Let *σ*^2^*c*_*n*_ be the vector of covariances between *x* and design points *ξ*, then we have , where  is the design matrix for the given model.

The classic kriging variance is  and the correction term in (A.3) equals to , where *V*_*ν*_ and

again depends on the derivatives (A.1) of the Matérn covariance function.
